# Comparison of unilateral and bilateral polymethylmethacrylate-augmented cannulated pedicle screw fixation for the management of lumbar spondylolisthesis with osteoporosis

**DOI:** 10.1186/s13018-020-01975-1

**Published:** 2020-09-29

**Authors:** Yao-yao Liu, Jun Xiao, Huai-jian Jin, Zhong Wang, Xiang Yin, Ming-yong Liu, Jian-hua Zhao, Peng Liu, Fei Dai

**Affiliations:** 1 Department of Spine Surgery, Army Medical Center of PLA, No. 10 Changjiang Road, Yuzhong District, Chongqing, 400042 People’s Republic of China; 2 Department of Orthopedics, Southwest Hospital of Army Medical University, PLA, No. 30 Gaotanyan Street, Shapingba District, Chongqing, 400038 People’s Republic of China

**Keywords:** Lumbar spondylolisthesis, Pedicle screw fixation, Polymethylmethacrylate augmentation, Osteoporosis

## Abstract

**Background:**

Cannulated pedicle screw (CPS) augmented by polymethylmethacrylate (PMMA) can achieve satisfactory clinical efficacy in the treatment of lumbar spondylolisthesis with osteoporosis. However, accurate application of CPSs will help to avoid the difficulty of screw revision and reduce the incidence of PMMA-related complications. This study aimed to investigate the mid-term efficacy of CPS compared to unilateral and bilateral applications in this common lumbar degenerative disease.

**Methods:**

May 2011 and May 2018, 50 patients with lumbar spondylolisthesis with osteoporosis who underwent posterior fixation and fusion using traditional pedicle screws or CPSs were included in the study. Patients were divided into two groups based on the application: the unilateral PMMA-augmented CPS group (UC, *n* = 29) and the bilateral PMMA-augmented CPS group (BC, *n* = 21). Operation time, blood loss, average hospitalization time, PMMA leakage, and other complications were recorded. The visual analog scale (VAS) and Oswestry disability index (ODI) scores were used to evaluate symptom recovery. Radiographic results were compared for intervertebral fusion and screw loosening.

**Results:**

There were no significant differences in the baseline data of the two groups.

The VAS and ODI scores improved significantly after surgery (*P* < 0.05), with no significant differences between the groups (*P* > 0.05). The operation time and blood loss in the UC group were significantly lower than those in the BC group (*P* < 0.05). However, the loss of intervertebral disk height and Taillard index did not differ significantly between the groups. The rates of PMMA leakage in the UC and BC groups were 7.0% and 11.9%, respectively (*P* < 0.05). Bony fusion was achieved in all groups without screw loosening at the last follow-up. Only one patient experienced superficial infection in both groups, while cerebrospinal fluid leakage was observed in two patients in the BC group.

**Conclusions:**

Unilateral application of PMMA-augmented CPS may provide adequate clinical safety and effectiveness in the surgical treatment of lumbar spondylolisthesis with osteoporosis.

## Background

Lumbar spondylolisthesis is a common spinal disease that presents as lower back pain, lower limb radiation pain, and intermittent claudication in elderly people. When conservative treatment is ineffective, surgical treatment for decompression, reduction, and reconstruction of spinal stability may be required. In lumbar spondylolisthesis, pedicle screw fixation is the main technique applied to maintain the stability and biomechanical characteristics of the spine [1]. However, the stability of the pedicle screw is greatly decreased in the lumbar spondylolisthesis cases that are combined with osteoporosis, which might lead to adverse events, such as screw loosening, extraction, or even breakage [2, 3], ultimately leading to failure of bony fusion.

Many researchers have attempted to solve this issue, and cannulated pedicle screw (CPS) augmented by polymethylmethacrylate (PMMA) is recognized as the most effective method developed to date [4, 5]. The primary focus has been on increasing stability by improving the side hole design of the screw [6, 7], as well as the optimization of the dose of PMMA in surgery [8, 9]. The safety and effectiveness of CPSs have been partially confirmed in previous studies [4, 5]. However, PMMA-related complications that can arise in clinical applications, such as PMMA leakage, allergic reactions, venous or pulmonary embolism, and difficulty in CPS revision, have drawn increasing attention from surgeons [10–13]. Therefore, clear guidance should be provided for a reasonable application to decrease the risk of these complications. To date, there is no consensus on the best application mode for PMMA-augmented CPSs. CPS fixation should meet the requirements for firm fixation, and the quantity used should be minimized. In brief, accurate and reasonable application of CPS fixation during surgery not only improves the effectiveness of surgery but also minimizes the risk of PMMA-related complications.

In the current study, we sought to investigate the mid-term efficacy of PMMA-augmented CPSs compared to unilateral and bilateral applications in lumbar spondylolisthesis with osteoporosis. To this end, we retrospectively reviewed the data of 50 consecutive patients treated with CPSs, and summarized the clinical outcomes and imaging findings of PMMA augmentation.

## Methods

### Patients

Between May 2011 and May 2018, 50 consecutive patients (12 males and 38 females) underwent transforaminal lumbar interbody fusion (TLIF) using CPSs for lumbar spondylolisthesis with osteoporosis. The inclusion criteria were as follows: patient age > 55 years; single-level lumbar spondylolisthesis (X-ray, degree I or II); T-score < −2.5 standard deviations (SDs) on dual-energy X-ray absorptiometry [9]; and no surgical contraindications. The exclusion criteria were as follows: allergy to the implant; normal bone mineral density (BMD); presence of other spine diseases; and infections, blood-related diseases, or other surgical contraindications. All patients were initially treated with conservative methods, but their lower back pain gradually progressed, resulting in neurological symptoms. None of the patients received medication for osteoporosis from local physicians before surgery.

The enrolled patients were divided into two groups according to the CPS application mode used in the treatment. The UC group, consisting of 29 patients (7 men and 22 women), with ages ranging from 57 to 80 years (mean, 71.8 ± 7.7 years), underwent TILF with unilateral CPS application. In this group, the BMD of the lumbar spine ranged from −2.5 to −4.4 SD, with a mean of −3.62 ± 0.7 SD. According to the Meyerding classification of spondylolysis [14], 19 cases had degree I, and 10 cases had degree II. The BC group included 21 patients (5 men and 16 women), with ages ranging from 56 to 82 years (mean, 68.4 ± 8.5 years). The T-score ranged from −2.5 to −4.7 SD, with a mean of −3.3 ± 0.6 SD. According to the Meyerding classification, 13 cases had degree I, and 10 cases had degree II. The general patient information is presented in Table 1. The study was approved by the Ethics Committee of Daping Hospital (IRB, 2019149). All methods were performed in accordance with relevant guidelines and regulations. All included patients provided informed consent.

**Table 1:** Baseline characteristics and clinical parameters of the 50 patients with lumbar spondylolisthesis with osteoporosis

	UC (*n* = 29)	BC (*n* = 21)	*P*
Sex (male: female)	7:22	5:16	0.651
Age (years)	71.8 ± 7.7	68.4 ± 8.5	0.728
Bone mineral density (T-score)	−3.6 ± 0.7	−3.3 ± 0.6	0.873
Surgical segment (L3:L4:L5)	1:15:13	1:9:10	0.635
Degree of displacement (I: II)	19: 10	13: 8	0.921
Operation time (min)*	186.1 ± 38.6	204.4 ± 27.1	0.034
Blood loss (ml)*	183.0 ± 23.6	236.4 ± 50.5	0.045
Hospitalization time (days)	5.5 ± 0.5	5.4 ± 0.7	0.098
Follow-up time (months)	29.1 ± 17.9	32.4 ± 15.7	0.234
Complications			
Superficial infection	1(3.4%)	1(4.8%)	0.387
PMMA leakage/total quantity of CPSs*	4/58(7.0%)	10/84(11.9%)	0.014
Cerebrospinal fluid leakage*	1(3.4%)	2(9.6%)	0.041
Fusion rate	100%	100%	0.173

*CPS* cannulated pedicle screw, *PMMA* polymethylmethacrylate, *UC* unilateral PMMA-augmented CPSs, *BC* bilateral PMMA-augmented CPSs

Values presented are the mean ± SD

*Significant if *P* < 0.05

### Surgical method

TLIF or minimally invasive TLIF is routinely performed. PMMA augmentation was performed in accordance with the surgeon’s manual findings during the surgery. Unilateral PMMA-augmented CPSs were used when the insertional torque during tapping was less than normal [15], and bilateral PMMA-augmented CPSs were added if the screw was not sufficiently stable. Patients in the UC group have CPSs implanted at the unilateral superior and inferior pedicles according to the surgeon’s decision during surgery, while the other side of the pedicles was implanted with traditional screws (Fig. 2). CPSs were implanted into all of the four pedicles on both sides in the BC group (Fig. 3). In particular, laminectomy was needed prior to PMMA injection so that the cement could be removed when PMMA leakage occurred. The PMMA powder and water agent were mixed, and then injected using a special device once the mixture had reached a dough-like consistency. The amount of PMMA (not more than 2 mL) was determined by intraoperative monitoring of intravertebral PMMA dispersion [16]. The surrounding tissue of the intervertebral disk space was loosened after the injection procedure was completed. Slipped vertebrae were reset by CPSs when the PMMA cement was completely hardened. A suitable cage was filled with crushed autologous bone for fusion, then rods were installed and nuts were locked. In this study, a new type of PMMA-augmented CPS, named “bone cement injectable cannulated pedicle screw (CICPS)” was developed by the authors for reduction and fixation, a detailed introduction of which was reported in detail in previous studies [17–20].

### Postoperative management

Patients were routinely treated with antibiotics to prevent surgical site infections in the first 24 h after surgery. Drainage tubes were removed when the amount of drainage fluid was less than 50 ml. Three days after surgery, all patients were encouraged to perform rehabilitation exercises by wearing a thoracolumbar brace for 3 months, and anti-osteoporosis treatment, including oral calcium 1200 mg/d and vitamin D 1200 iu/d, were supplemented as soon as possible after surgery. Additionally, female patients (creatinine clearance rate ≤ 36%) were scheduled to receive bisphosphonates intravenously once a year.

### Radiographic and clinical assessments

**Fig. 1:**
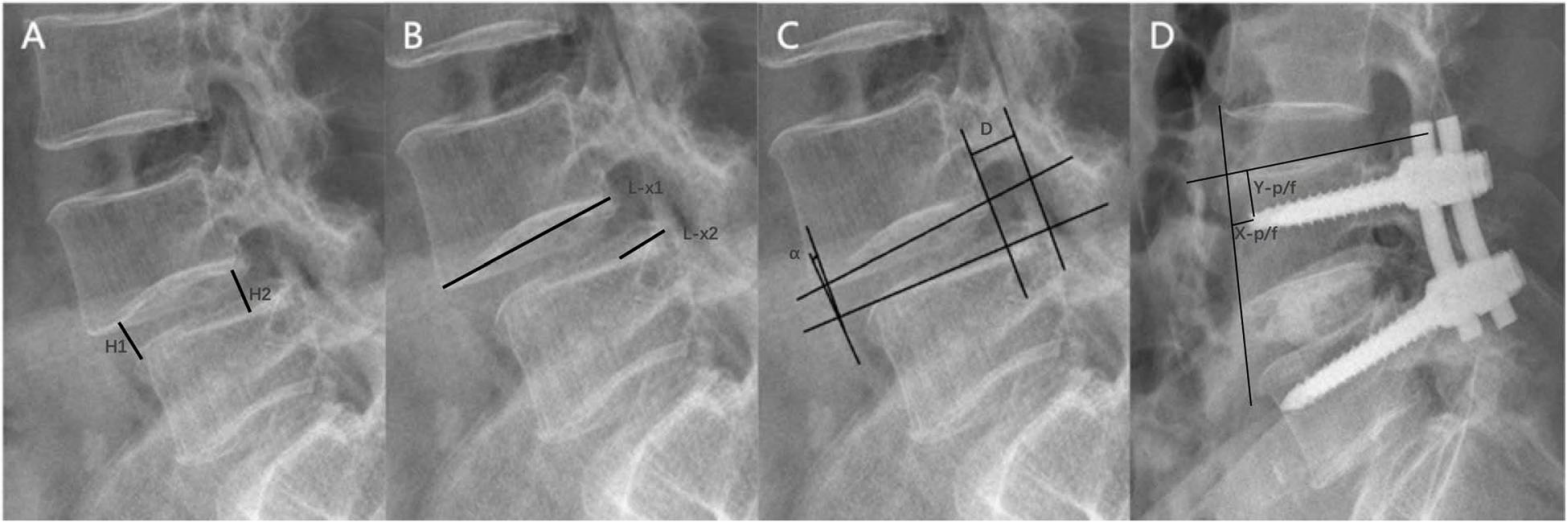
Measurement methods for intervertebral disk height (**a**, H1/2 + H2/2), Taillard index (**b**, L-x2/L-x1), vertebral movement (**c**, D_extension_-D_flexion_ and α_extension_-α_flexion_), and screw displacement values (**d**)

Operation time, blood loss, and hospitalization time were recorded to evaluate the basic condition of the surgery. A review was carried out at 3, 6, and 12 months after surgery, and every 6 months thereafter. Moreover, lumbar X-ray films were obtained to evaluate bony fusion, screw loosening, or pull-out-related imaging indicators, including intervertebral disk height and screw displacement. Intervertebral disk height was defined as the average distance between the anterior and posterior edges of the vertebral body and the endplates (H1/2 + H2/2) (Fig. 1a) [19]. The Taillard index was determined to assess the degree of vertebral body slipping (L-x2/L-x1) (Fig. 1b) [21]. Visual analog scale (VAS) and Oswestry disability index (ODI) scoring systems were used to evaluate pain and functional recovery in the lower limbs, respectively. Complications, such as wound infection, cerebrospinal fluid leakage, and PMMA leakage, were recorded.

*Evaluation criteria for spinal fusion on X-ray films* [19, 22]: (1) Passage of trabecular bone through the bone graft area; (2) vertebral movement between the flexion and extension X-ray film < 3 mm (*D*_extension_-*D*_flexion_) (Fig. 1c), or change in the intervertebral space angle < 5° (α_extension_-α_flexion_) (Fig. 1c); and (3) bone growth through the intervertebral disk space.

*Method of measuring screw displacement*: Loosening and displacement of the screws were reflected by the distances from the screw tip to the anterior margin (*X*) and superior endplate (*Y*) of the vertebral body (Fig. 1d). Compared to the postoperative value (*X*-*p*, *Y*-*p*), a 1 mm displacement in the screw-bone interface at the final follow-up (*X*-*f*, *Y*-*f*) was defined as screw loosening, as described by Moon et al. [23].

All measurements were made by the same orthopedic surgeon with extensive experience in spine surgery. The mean of three measurements, obtained at different time points, with 2-week intervals, was determined to reduce measurement error.

### Statistical analysis

The SPSS 25.0 statistical software (SPSS, Inc., Chicago, IL, USA) was used for all statistical analyses. All measurement data are expressed as mean ± SD. Pre- and postoperative measurement data were compared using a paired *t* test. Statistical analyses between the two groups were performed using the chi-square test or Fisher’s exact test for count data, and Student’s *t* test for measurement data. *P* values < 0.05 were considered to indicate statistical significance.

## Results

There were no statistically significant differences in the age, sex, displaced segment, degree of displacement, and BMD between the two groups (Table 1). The follow-up period ranged from 6 to 96 months, with a mean of 29.1 months in the UC group and 32.4 in the BC group. The operation time in the UC group (186.1 ± 38.6 min) was significantly lower than that in the BC group (204.4 ± 27.1 min; *P* < 0.05). Blood loss in the UC group (183.0 ± 23.6 mL) was significantly lower than that in the BC group (236.4 ± 50.5 mL; *P* < 0.05). There was no significant difference in the hospitalization time between the UC group (5.5 ± 0.5 days) and the BC group (5.4 ± 0.7 days; *P* > 0.05). In the UC group, 58 CPSs were implanted in 29 patients, and PMMA leakage during surgery occurred in four screws, with an incidence of 7.0%. In the BC group, 84 CPSs were implanted in 21 patients, and ten of PMMA leakage occurred in 11.9% of patients. No serious complications, such as nerve injury or pulmonary embolism, were observed in any of the PMMA-leakage cases. One superficial infection was noted in each group, which was controlled by intravenous administration of antibiotics. Cerebrospinal fluid leakage occurred in one patient in the UC group, and two patients in the BC groups; all cases healed completely after the drainage tube was removed after 2 weeks of bed rest.

The VAS and ODI scores immediately after surgery and at the final follow-up were significantly lower than the respective preoperative readings in both groups (*P* < 0.05). In both groups, the VAS and ODI scores at the final follow-up were significantly lower than those immediately after the operation (*P* < 0.05). However, no significant difference in VAS or ODI was found between the two groups, either postoperative or at final follow-up (*P* > 0.05). The data of patients in both groups are shown in Tables 2 and 3.

**Table 2:** Comparison of the VAS between 2 groups preoperatively, immediately after surgery, and at final follow-up

Group	*n*	Preoperatively	Immediately after surgery	Final follow-up
UC	29	8.4 ± 1.1	2.5 ± 0.7*	2.2 ± 0.8*, **
BC	21	8.0 ± 0.8	3.0 ± 0.4*	2.8 ± 0.5*, **, ***
Statistics		*t* = 0.245, *P* = 0.756	*t* = 4.253, *P* = 0.076	*t* = 1.723, *P* = 0.546

*VAS* visual analog scale, *UC* unilateral PMMA-augmented CPSs, *BC* bilateral PMMA-augmented CPSs

Values presented are the mean ± SD

Significant if *P* < 0.05

*P < 0.05 vs. preoperatively values

**P < 0.05 vs. immediately after surgery values

***P > 0.05 vs. final follow-up values of the UC group

**Table 3:** Comparison of the ODI between 2 groups preoperatively, immediately after surgery, and at final follow-up

Group	*n*	Preoperatively	Immediately after surgery	Final follow-up
UC	29	51.9 ± 10.4	10.0 ± 6.1*	9.8 ± 0.8*, **
BC	21	54.0 ± 10.9	11.3 ± 0.4*	10.5 ± 0.5*, **, ***
Statistics		*t* = 0.717, *P* = 0.395	*t* = 5.143, *P* = 0.176	*t* = 1.003, *P* = 0.246

*ODI* Oswestry disability index, *UC* unilateral PMMA-augmented CPSs, *BC* bilateral PMMA-augmented CPSs

Values presented are the mean ± SD

Significant if *P* < 0.05

*P < 0.05 vs. preoperatively values

**P > 0.05 vs. immediately after surgery values

***P > 0.05 vs. final follow-up values of the UC group

**Fig. 2:**
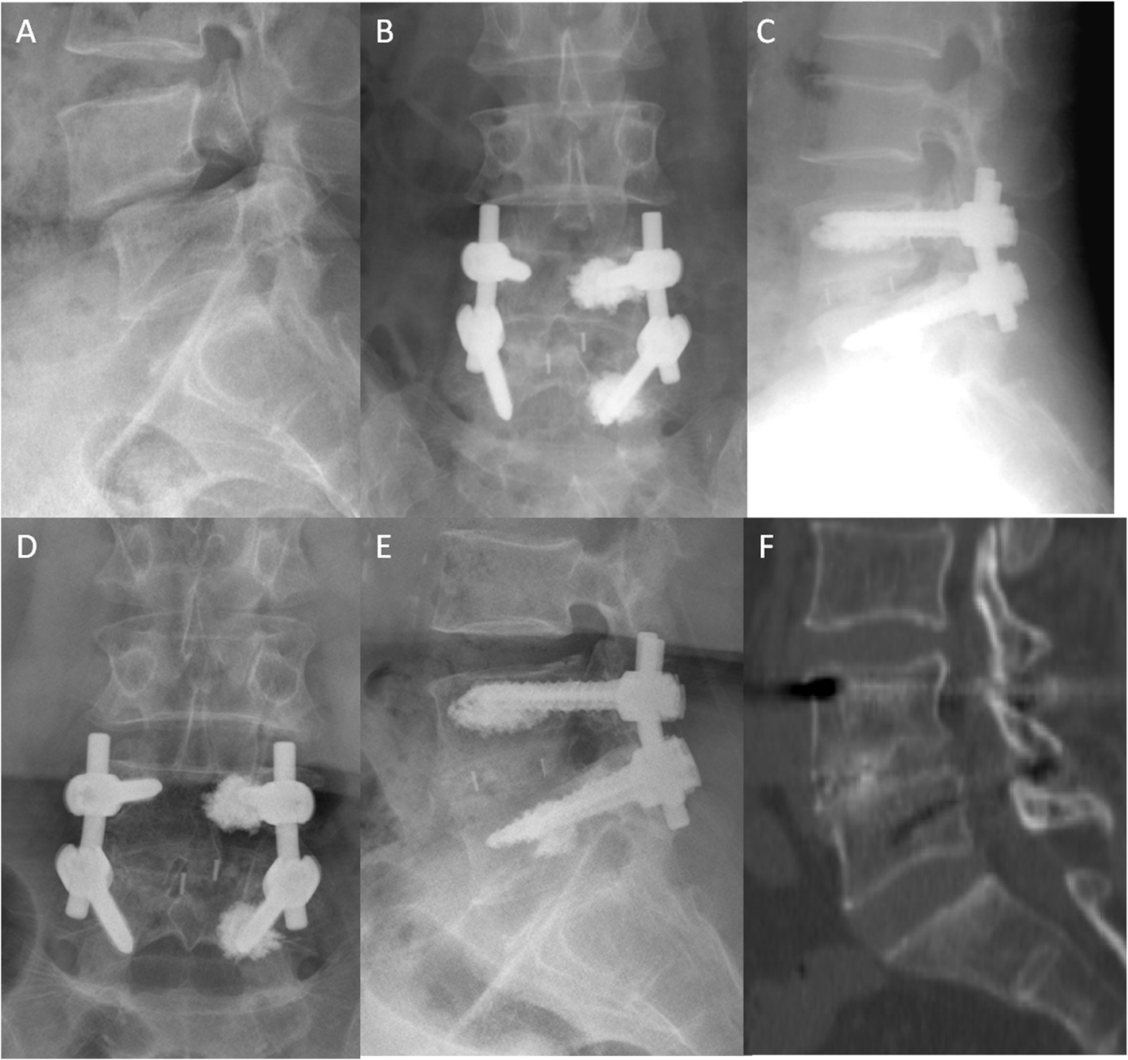
A 65-year-old female diagnosed with spondylolysis at the L4 vertebral body with osteoporosis (*T* = −3.2). **a** Preoperative lateral X-ray showing grade I lumbar spondylolisthesis. **b**–**c** Unilateral PMMA-augmented CPSs are used for spinal fixation. Immediate postoperative radiographs showing reconstruction for spondylolisthesis without PMMA leakage. **d**–**e** CPSs are observed in place after 49 months of surgery. **f** CT scan showing that bony fusion was achieved

**Fig. 3:**
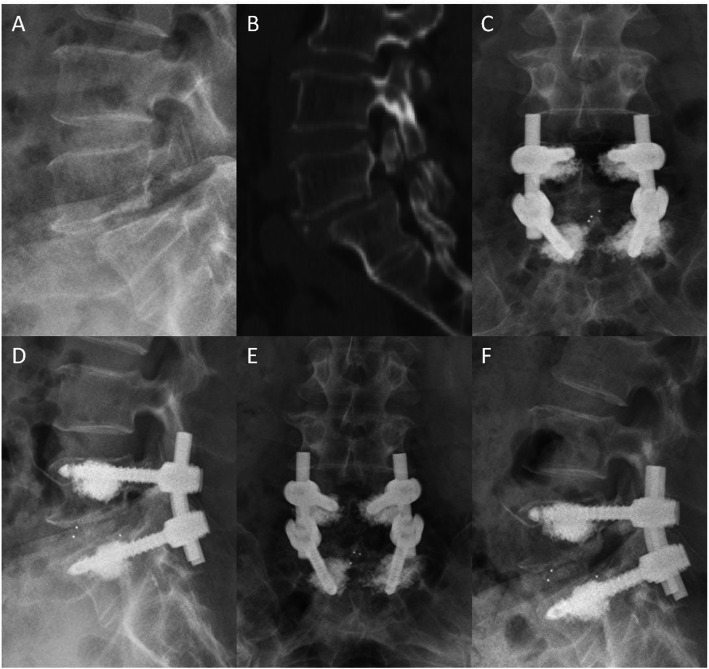
A 72-year-old female diagnosed with lumbar spondylolysis at the L5 vertebral body with osteoporosis (*T* = −2.8). **a**–**b** Preoperative lateral X-ray and CT scan showing grade II ture spondylolisthesis. **c**–**d** Bilateral PMMA-augmented CPSs are used for spinal fixation. The L5 vertebral body is well corrected, but PMMA leaked into the vertebral vein without any PMMA-related symptoms. **e**–**f** Lateral X-ray and CT scan at the last follow-up showing that no screw loosening occurs, and bony fusion is achieved. Lower back pain is ameliorated for this patient

Illustrative cases of the UC and BC groups are shown in Figs. 2 and 3. The intervertebral disk height and degree of spondylolisthesis (Taillard index) in the two groups were significantly restored after surgery (*P* < 0.05). The intervertebral disk height and Taillard index were not statistically significant as time went by (*P* = 0.672). Moreover, there was no significant difference in correction loss between the two groups at the last follow-up (*P* = 0.289) (Table 4). Furthermore, the displacement distances (*X* and *Y*) after surgery and at the last follow-up were not significantly different between the two groups (*P* > 0.05). The absolute values of the differences in the *X* (*X*-*f* minus *X*-*p*) and *Y* (*Y*-*f* minus *Y*-*p*) were less than 1 mm for all patients in both groups, indicating that no screw loosening was observed (Table 4).

**Table 4:** Radiographic characteristics in the 2 groups

Group	UC (*n* = 29)	BC (*n* = 21)	*P*
Intervertebral disk height (mm)			
Preoperatively	9.1 ± 3.9	8.9 ± 2.4	0.046
Immediately after surgery*	14.1 ± 2.4	13.5 ± 3.6	0.192
Final follow-up*, **	13.5 ± 3.0	12.8 ± 2.5	0.098
Loss of correction	1.4 ± 0.8	1.9 ± 0.9	0.672
Taillard index (%)			
Preoperatively	29.8 ± 10.4	30.5 ± 9.1	0.958
Immediately after surgery*	7.4 ± 6.3	6.8 ± 7.8	0.091
Final follow-up*, **	7.0 ± 5.9	6.5 ± 7.9	0.193
Loss of correction	0.3 ± 0.4	0.3 ± 0.2	0.289
Screw displacement (mm)			
*X*-*p*	8.2 ± 4.4	7.4 ± 6.3	0.093
*X*-f**	8.3 ± 4.7	7.8 ± 4.1	0.321
*Y*-*p*	8.5 ± 2.9	8.9 ± 4.7	0.447
*Y*-f**	8.1 ± 1.8	8.1 ± 5.1	0.632

*X* distance between the screw tip and the anterior surface of the vertebral body, *Y* distance from the screw tip to the superior endplate of the vertebral body, *-p* immediately after surgery, *-f* final follow-up, *UC* unilateral PMMA-augmented CPSs, *BC* bilateral PMMA-augmented CPSs

Values presented are the mean ± SD

Significant if *P* < 0.05.

*P < 0.05 vs. preoperatively values

**P > 0.05 vs. immediately after surgery values

## Discussion

The biomechanical stability of pedicle screw fixation systems is particularly important in patients with lumbar spondylolisthesis and osteoporosis [11, 24]. Studies have shown that the use of PMMA-augmented CPSs to reconstruct the displaced vertebral body and perform bony fusion is still the main surgical method for these patients [19, 20, 24–28] (Fig. 3). Theoretically, more CPSs require a greater holding force by internal fixation; however, overuse of CPSs may increase the risk of complications related to PMMA leakage, including allergic reactions, venous or pulmonary embolism, and difficulty with revision. There are very few reports on the accurate and reasonable application guidelines of CPSs to improve the rationality in clinical practice. We reviewed the published literature and found that some studies used CPSs on bilateral sides [25–28], while others used only the unilateral side [17, 19, 20]. Therefore, it is clinically important to explore whether unilateral PMMA-augmented CPSs can provide stability that is equal to, or better than that of bilateral PMMA augmentation. In addition, it is important to determine any differences in the effectiveness and complications between the two methods.

In the current study, the CPSs augmented by PMMA, either unilaterally or bilaterally, could improve the reduction in the postoperative slip degree. This finding was based on the significant differences in the intervertebral disk height and Taillard index preoperatively and postoperatively in both groups. During follow-up, intervertebral disk height is a key indicator for treatment success, and previous studies have confirmed that reduction can restore physiological alignment and balance, especially for high-grade spondylolisthesis [29, 30]. Furthermore, Chalee-Valayer et al. [31] and Roussouly et al. [32] reported that loss of intervertebral disk height was positively correlated with lower back pain. In the UC and BC groups in the current study, the mean intervertebral disk height was lost at the last follow-up, which is consistent with the literature [27]. However, this change was not statistically significant compared to immediately after surgery, and the clinical symptoms of the patients were not aggravated by this loss; this phenomenon can be explained by physiological progress. Furthermore, interbody fusion cages are possible to sink after surgery because of osteoporosis. In the current study, unilateral and bilateral fixations were equally effective at maintaining disk height, as demonstrated by comparing the loss of intervertebral space height between the UC and BC groups.

The Taillard index is another key indicator for evaluating the maintenance of spinal reduction. Floman et al. [33] and Goyal et al. [34] suggested that the displaced vertebral body should be anatomically restored as much as possible for patients with lumbar spondylolisthesis, so as to increase the area of intervertebral fusion. Kim et al. [35] and Wang et al. [36] reported that CPSs were better able to restore displaced vertebral bodies than traditional screws. Similarly, our results showed that PMMA-augmented CPSs could avoid vertebral body slipping, and unilateral and bilateral fixations both showed long-term maintenance of spinal stability after surgery.

Previous studies revealed that the screw loosening rate was increased in patients with osteoporosis, which might lead to serious consequences, such as screw fracture, non-fusion, and pseudarthrosis [37–40]. However, no screw loosening was observed in the current study, as confirmed by screw displacement less than 1 mm at the last follow-up in all cases. However, the incidence of complications related to PMMA is known to increase with the amount of PMMA used in a single vertebral body; this implies that bilateral PMMA-augmented CPSs have a greater risk of PMMA leakage. In fact, the PMMA-leakage rate of CPSs differed greatly among previous studies, and Angel et al. [25] and Wang et al. [27] reported that the rate was in the range of 29.3–36.1% for bilateral augmentation. In the present study, the rate was 11.9%, which was lower than that reported in previous studies; this may be related to different designs of CPSs used in different studies. However, the leakage rate for unilateral augmentation was 7% in the UC group, which was significantly lower than that in the BC group. Unilateral CPSs may reduce the risk of PMMA leakage by reducing the amount of PMMA used.

The biomechanical properties of the vertebral body after surgery have also attracted the attention of researchers. Baroud et al. [41] and Uppin et al. [42] demonstrated that PMMA augmentation increased the fracture risk for the vertebral body or the adjacent ones. In the present study, no significant fractures were observed during follow-up, which could be related to the small number of patients enrolled or the relatively short follow-up period. Indeed, the effects of alterations to biomechanical properties are sometimes difficult to observe in the short term, although they may be obvious in the long term.

Singh et al. [43] performed a systematic analysis of a PMMA-augmented CPS. Their findings indicated that the average VAS score before operation was 8.4 (range, 8–9.2) compared to 2.3 (range, 1.42–4.8) at the last follow-up. Moreover, for assessment of functional recovery, the average improvement in the ODI was 42.1. These results were in line with those of the current study, in which the VAS and ODI scores significantly improved immediately after surgery and at the last follow-up (*P* < 0.05) compared to those before surgery in both groups. Additionally, there were significant differences in VAS and ODI scores immediately after surgery and at the final follow-up (> 6 months after surgery) (*P* < 0.05). These results indicate that satisfactory mid-term clinical outcomes can be achieved in both groups.

The operation time, blood loss, and cerebrospinal fluid leakage in the UC group were significantly lower than those in the BC group (*P* < 0.05), demonstrating that unilateral PMMA-augmented CPSs are less invasive and can be performed less time than bilateral CPSs; these factors are especially important for elderly patients with comorbidities. Because lumbar spondylolisthesis usually occurs in adults older than 50 years, the patients in this study were older and may have had many comorbidities and severe osteoporosis; thus, complex surgical methods could not be tolerated by these patients.

This study has several limitations that should be considered. First, the measurement method cannot accurately demonstrate the changes at the screw tip. Second, the analysis can also be subjected highly to individual variants, which is not tested by different radiologists due to the projection or obliquity of the X-ray view. In this context, a computed tomography (CT) scan would be superior to analyze the evidence of screw loosening, and provide a more robust conclusion. Finally, the study was a retrospective study with defects in the study design, and the sample size of this study was relatively small, which reduced the credibility of the study.

## Conclusions

Both unilateral and bilateral applications of CPSs are clinically safe and effective methods to augment pedicle screws in patients with lumbar spondylolisthesis and osteoporosis. However, unilateral PMMA-augmentation has the advantages of reduced blood loss, operative time, and complications in elderly patients with comorbidities. This study could provide an evidence-based basis for developing guidelines for CPS application, especially in patients with lumbar spondylolisthesis and osteoporosis.

## Data Availability

The datasets generated and analyzed during the present study are available from the corresponding author on reasonable request.

## Abbreviations

BMD: Bone mineral density
CICPS: Bone cement-injectable cannulated pedicle screw
CPS: Cannulated pedicle screw
CT: Computed tomography
LSD: Least significant difference
MRI: Magnetic resonance imaging
ODI: Oswestry disability index
PMMA: Polymethylmethacrylate
SD: Standard deviation
TLIF: Transforaminal lumbar interbody fusion
VAS: Visual analog scale
